# Identifying epileptogenic abnormalities through spatial clustering of MEG interictal band power

**DOI:** 10.1002/epi4.12767

**Published:** 2023-06-05

**Authors:** Thomas W. Owen, Vytene Janiukstyte, Gerard R. Hall, Jonathan J. Horsley, Andrew McEvoy, Anna Miserocchi, Jane de Tisi, John S. Duncan, Fergus Rugg‐Gunn, Yujiang Wang, Peter N. Taylor

**Affiliations:** ^1^ CNNP Lab, Interdisciplinary Computing and Complex BioSystems Group, School of Computing Newcastle University Newcastle upon Tyne UK; ^2^ UCL Queen Square Institute of Neurology London UK; ^3^ National Hospital for Neurology & Neurosurgery London UK; ^4^ NIHR University College London Hospitals Biomedical Research Centre, UCL Queen Square Institute of Neurology London UK; ^5^ Faculty of Medical Sciences Newcastle University Newcastle upon Tyne UK

**Keywords:** clustering, epilepsy, MEG, outcome, prediction, surgery

## Abstract

Successful epilepsy surgery depends on localizing and resecting cerebral abnormalities and networks that generate seizures. Abnormalities, however, may be widely distributed across multiple discontiguous areas. We propose spatially constrained clusters as candidate areas for further investigation and potential resection. We quantified the spatial overlap between the abnormality cluster and subsequent resection, hypothesizing a greater overlap in seizure‐free patients. Thirty‐four individuals with refractory focal epilepsy underwent pre‐surgical resting‐state interictal magnetoencephalography (MEG) recording. Fourteen individuals were totally seizure‐free (ILAE 1) after surgery and 20 continued to have some seizures post‐operatively (ILAE 2+). Band power abnormality maps were derived using controls as a baseline. Patient abnormalities were spatially clustered using the *k*‐means algorithm. The tissue within the cluster containing the most abnormal region was compared with the resection volume using the dice score. The proposed abnormality cluster overlapped with the resection in 71% of ILAE 1 patients. Conversely, an overlap only occurred in 15% of ILAE 2+ patients. This effect discriminated outcome groups well (AUC = 0.82). Our novel approach identifies clusters of spatially similar tissue with high abnormality. This is clinically valuable, providing (a) a data‐driven framework to validate current hypotheses of the epileptogenic zone localization or (b) to guide further investigation.

## 1 INTRODUCTION

Neurosurgery is a treatment option for individuals with refractory focal epilepsy. Surgical success relies on the accurate delineation and resection of the epileptogenic zone (EZ), an area of tissue needed to generate seizures.^1^ At present, localization of the EZ typically uses visual analysis of structural and functional imaging data. In recent years, numerous studies have developed quantitative markers of the EZ.^2^, ^3^, ^4^, ^5^, ^6^, ^7^ Both qualitative and quantitative approaches may identify multiple widespread abnormalities in a patient, leading to uncertainty over the design of focal surgery. Thus, in considering abnormalities for further investigation, their spatial proximity should be taken into account.

One approach to develop quantitative markers of the EZ involves neurophysiological abnormality mapping.^4^, ^7^, ^8^, ^9^ This approach posits that regions with abnormal neural dynamics (eg, measured using band power), relative to normative baselines, should be targeted by surgery to achieve seizure freedom.^4^, ^7^, ^9^ A key limitation of such studies is the potential for false‐positive abnormalities, which may be far from the EZ, and could lead to mislocalization and poor outcomes.^7^

We developed a framework to account for neurophysiology abnormalities and their spatial proximity. We use clustering techniques to group areas that are both highly abnormal and spatially similar. We hypothesized a greater overlap between the abnormal cluster and subsequent resection in patients who were seizure‐free after surgery. Second, we hypothesize that identifying clusters of abnormality has clinical value to assist the localization of the EZ.

## 2 METHODS

### 2.1 Patients and abnormality mapping

Resting‐state MEG recordings were acquired for 34 refractory neocortical patients and 70 healthy controls using a 275‐channel whole head CTF MEG scanner. Individual subject T1‐weighted MRI was performed using a 3T GE Signa HDx scanner. Fourteen patients were entirely seizure‐free (ILAE 1) 1 year post‐operatively.^10^ Patient‐specific maps of eyes‐closed interictal band power abnormalities were constructed using healthy data as a baseline. Regional abnormalities in five frequency bands (delta; 1‐4 Hz, theta; 4‐8 Hz, alpha; 8‐13 Hz, beta; 13‐30 Hz, gamma; 30‐47.5 Hz and 52.5‐80 Hz) were estimated and downsampled by taking the maximum absolute *z*‐score across frequencies. A complete description of data acquisition, pre‐processing, and abnormality mapping has been outlined previously.^7^ Subject data and clustering results are summarized in Table S1.

### 2.2 *K*‐means clustering

We used *k*‐means clustering to account for the spatial similarity of regions when proposing tissue for resection. *K*‐means is an unsupervised clustering technique that assigns observations into *K* clusters, minimizing the intra‐cluster sum of squared distances. Four features were selected to cluster the data. The first three correspond to the standard space *x*, *y*, and *z* coordinates of each neocortical region of interest (ROI) centroid. The fourth feature corresponds to the band power abnormality within each ROI. All four features were mean‐centered and scaled by the standard deviation to minimize the bias when computing measures of cluster distance. Once partitioned into *K* clusters, the cluster containing the most abnormal neocortical region was chosen for further investigation. Finally, we constrained the cluster to a single hemisphere by retaining only regions in the most common hemisphere. Regions were constrained to a single hemisphere to better reflect focal resections, which would not span multiple hemispheres. These methods provide a data‐driven approach to propose a spatially constrained cluster, which can be considered for resection or further investigation. Note that this approach means that different patients can have different‐sized clusters, which conforms to the clinical practice of different patients with different resection sizes.

In addition to the number of clusters (*K*), we varied the number of strongest cortical abnormalities (N) used to identify the clusters of abnormality. The optimal parameter set was chosen using a leave‐one‐out procedure. The parameter pair that maximized the separability of surgical outcome groups in the remaining 33 patients was chosen as the optimal for the hold‐out patient. The top N abnormalities were chosen over a set *z*‐score threshold as the magnitudes of abnormalities in patients have been shown to relate to the duration of epilepsy^9^, ^11^ and surgical outcome.^7^ Candidate values of *K*, 2‐4, were selected based on visual inspection of patient scree‐plots and the N strongest abnormalities were thresholded between 30 and 70 as this range provides a good balance between sensitivity and specificity.

### 2.3 Validation of the abnormality cluster

**FIGURE 1:**
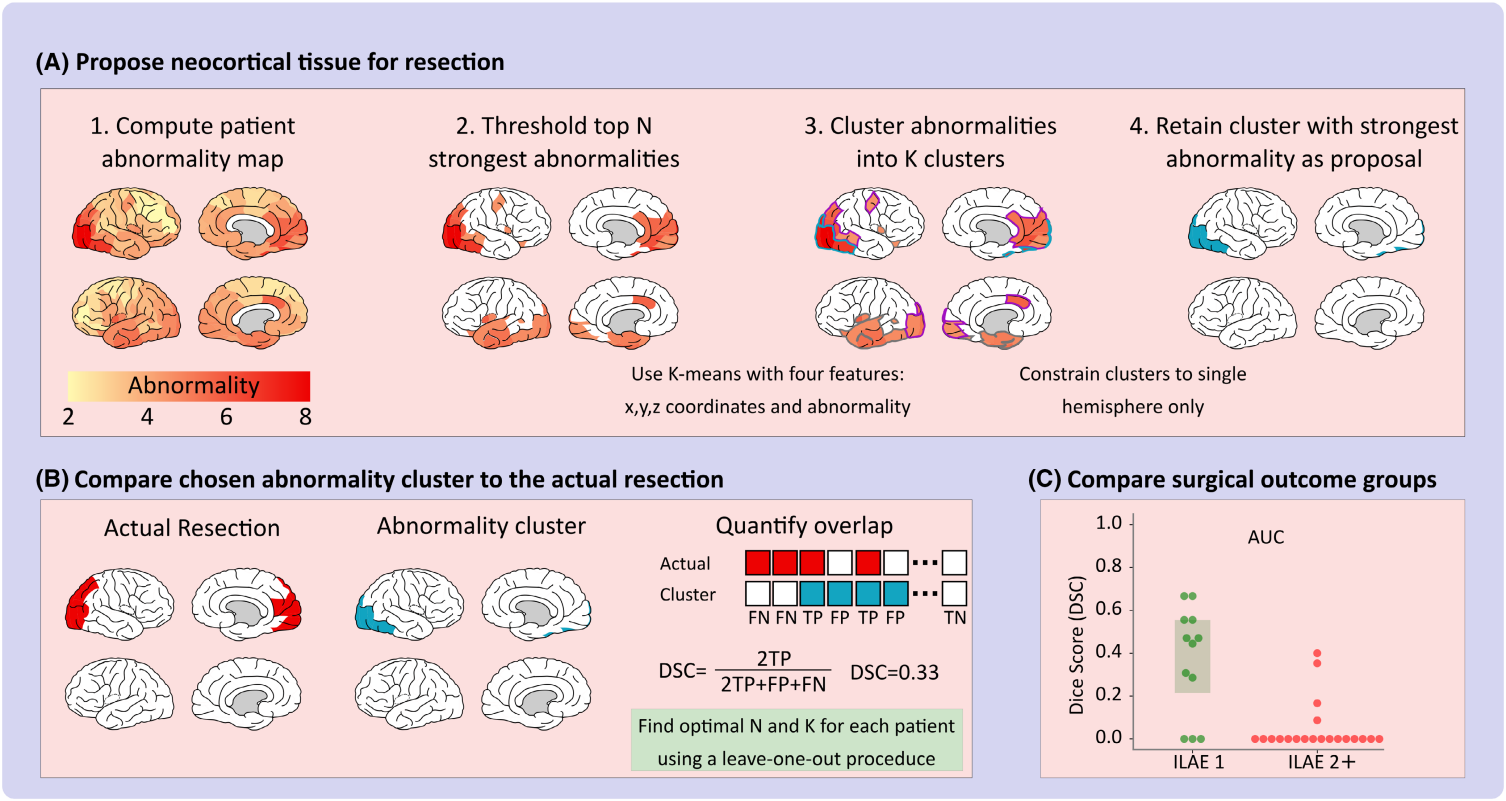
Processing pipeline to identify abnormality clusters. A, First, patient abnormality maps were constructed using interictal band power MEG recordings and healthy recordings as a baseline. Second, patient abnormality maps are filtered to retain only the top N strongest abnormalities. Third, the strongest abnormalities are grouped into *K* clusters using *k*‐means clustering with four features (*x*, *y*, *z* coordinates of each ROI and the abnormality value). Each feature is scaled to minimize any bias in the clustering. Finally, the cluster containing the most abnormal region is selected, with the added constraint that regions reside in a single hemisphere to better reflect focal resections. The chosen hemisphere was decided based on which contained over half of the regions within the cluster. B, The overlap between the abnormality cluster and actual resection was compared using the dice score (DSC) which measures the ratio of overlap (2TP) relative to the union of the abnormality cluster and resection (2TP + FP + FN). FN, false negative; FP, false positive; TN, true negative; TP, true positive. The DSC varies from 0 to 1 with DSC = 0 corresponding to no overlap at all, and DSC = 1 corresponding to the perfect overlap (identical regions). Steps (A) and (B) are repeated to find the optimal values of N and *K* to maximize the DSC across the cohort whilst balancing model complexity. C, The DSC across the whole cohort is compared with differences between seizure‐free (ILAE 1) and not‐seizure‐free (ILAE 2+) quantified using the area under the receiver operator curve (AUC).

The overlap between the abnormality cluster and the resection was quantified using the dice score (DSC). The DSC, defined in Equation 1, is the ratio of the overlap between the abnormality cluster and the subsequent resection (true positive; 2TP) relative to the union of the two (2TP + FP + FN). Ranging from zero to one, smaller dice scores correspond to a lower overlap between the abnormality cluster and resection, whilst larger dice scores correspond to a higher overlap. Finally, we used the area under the receiver operator curve (AUC) to quantify the separability of surgical outcome groups based on their corresponding dice scores. A one‐tailed Mann‐Whitney *U* test was performed to assess the significance of the surgical outcome group separability. The identification of clusters and quantification of the overlap between the abnormality cluster and resection are illustrated in Figure 1.

(1) $$\mathrm{DSC}=\frac{2\mathrm{TP}}{2\mathrm{TP}+\mathrm{FP}+\mathrm{FN}}.$$

## 3 RESULTS

Using pre‐operative interictal MEG band power abnormality maps we sought to propose plausible areas of high abnormality that could contain the EZ while accounting for the spatial proximity of neocortical regions. Two clustering parameters were tuned for each patient using a leave‐one‐out approach which maximizes the separability of surgical outcome groups in the remaining patients. The optimal clustering parameters were identical for all patients and were identified as the top 50 strongest neocortical abnormalities (N), partitioning into three clusters (*K*) based on the results of the leave‐one‐out procedure.

**FIGURE 2:**
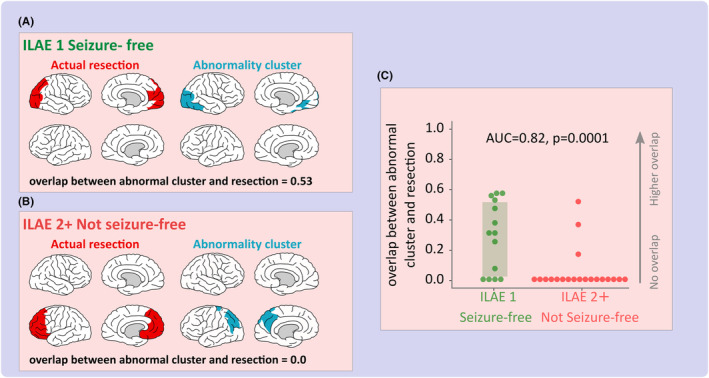
Overlap between the abnormality cluster and actual resection. A, Illustration of the overlap between the abnormality cluster and actual resection in an example seizure‐free patient. Visually, a high overlap exists between the actual resection (red) and the abnormality cluster (blue). This is apparent with a DSC = 0.53 indicating a good overlap. In this scenario, we hypothesized a good surgical outcome as both resection masks cover similar tissue. Conversely, (B) corresponds to an example poor outcome patient with no overlap between the abnormality cluster and the resection. A DSC = 0 would suggest a poor outcome for this patient as the abnormality cluster and the resection are in complete disagreement. Cohort‐wide comparisons (C) demonstrate that in seizure‐free patients (green) as hypothesized there is a higher overlap between the abnormality cluster and the subsequent resection in contrast to patients with poor surgical outcomes (red), AUC = 0.82, *P* = 0.0001.

Two example patients illustrate the difference between surgical outcome groups (Figure 2A,B). For the seizure‐free patient, there is a high overlap between the abnormality cluster and the subsequent surgical resection in the occipital lobe (Figure 2A). This overlap is quantified with a dice similarity of 0.53. Conversely, for the poor outcome patient, there is no overlap between the abnormality cluster and the resection, with the abnormality cluster localized in a different lobe (Figure 2B). These results suggest that our data‐driven clustering of abnormalities may provide localizing information.

Expanding the analysis to the full cohort (Figure 2C) it is evident that stronger dice scores are attributed to a positive surgical outcome. Indeed, 71% of ILAE 1 patients had some overlaps, suggesting even partial resection of our abnormality cluster predisposes a high chance of seizure freedom. Furthermore, 85% of patients with persistent seizures (ILAE 2+) had zero overlap, suggesting potential mislocalization in those patients. In addition, this effect successfully discriminated outcome groups (AUC = 0.82, *P* = 0.0001). Individuals with overlap between the abnormality cluster and actual resection were 14 times more likely to be seizure‐free post‐operatively at 12 months (Odds Ratio: 14.2, 95% confidence interval: [2.6, 76.7]). Similar results were found for other measures of overlap (see Appendix S1). Moreover, the overlap between the proposed cluster of abnormality and resection was associated with long‐term seizure freedom (AUC = 0.70, 12 ILAE 1, 22 ILAE 2+). Together, our results suggest that clustering the strongest patient‐specific abnormalities may provide clinically useful proposals of tissue that may contain the EZ.

## 4 DISCUSSION

In this study, we introduced a novel framework to identify spatially proximal abnormality clusters. We demonstrated that abnormality clusters overlap with the subsequent resection in almost all seizure‐free patients. In contrast, most patients who were not seizure‐free had resections that were completely discordant with the abnormality cluster. We suggest that abnormality clusters could be clinically useful to assist in the delineation of the cortex to be resected or investigated further to cure drug‐resistant focal epilepsy.

Our current study could complement the existing literature that develops markers of the EZ using structural^2^, ^12^, ^13^ and functional^4^, ^7^, ^14^, ^15^, ^16^ data. The clinical adoption of previous work is difficult since markers of the EZ may neglect the spatial properties of the tissue. As such, proposals of which tissue to resect may not be spatially contiguous, with abnormalities spanning multiple lobes or hemispheres. The benefits of accounting for spatial properties are well established in neuroimaging using, eg, random field theory.^17^ Random field theory has been applied in the context of epilepsy,^18^ with studies identifying clusters of statistically abnormal voxels in patients with epileptogenic malformations such as focal cortical dysplasia. In the context of surgical resections, we incorporated the spatial proximity of cortical abnormalities using *k*‐means clustering, demonstrating that simple clustering techniques could be applied to current markers of epileptogenic tissue in order to suggest realistic data‐driven areas of tissue for resection.

It has been established that the resection of epileptogenic tissue is associated with seizure freedom post‐surgically.^1^ A stronger overlap between the abnormality cluster and resection in seizure‐free patients suggests that our abnormality clusters may indeed be capturing the EZ. Interestingly, even in patients rendered seizure‐free, the overlap is not perfect (Figure 2). The discrepancies between the abnormality cluster and resection could be attributed to a number of factors. First, targeting the epileptogenic tissue for resection may require that healthy tissue is also removed, tissue that is not currently considered within our framework. As such, this would increase the false negative rate, thus decreasing the dice overlap. Alternatively, a decrease in the overlap could be attributed to a difficulty to localize EZ, either due to inconsistencies across modalities or an extensive epileptogenic network.^9^ As a result, a larger area of tissue may be targeted to ensure the total resection of the EZ. This may have been the case in Figure 2A with the resection including the inferior and superior parietal lobe. Finally, the suggested abnormality cluster may contain abnormal tissue that visually appears normal in neuroimaging and neurophysiology modalities such as MRI and MEG. For example, the abnormality cluster in Figure 2A extends to the inferior temporal lobe, areas of tissue that were not targeted during the resection. The results of this study suggest that the abnormality cluster contains complementary information which could aid in the accurate lateralization and localization of the EZ.

One limitation of our current clustering framework is the added constraint that abnormalities should reside in a single hemisphere. Although the constraint of single hemisphere clusters better reflects a focal resection, it is conceivable that the strongest abnormalities could span both hemispheres. As such by constraining abnormalities to a single hemisphere we are discarding potentially valuable information that may alter clinical decision making. Future studies could investigate whether intracranial EEG implantation is associated with MEG band power abnormalities and determine if the cluster of strongest abnormalities provides complementary information to guide electrode implantation in seemingly healthy tissue across both hemispheres.

The accurate delineation and resection of the EZ have been shown to be associated with surgical success. We introduce a fully data‐driven clustering technique to spatially constrain markers of the EZ into plausible clusters of abnormality. Our approach could be clinically valuable, offering a data‐driven cluster of tissue believed to contain the EZ. This can be used for validation against the current candidate tissue for resection, or to guide further investigation.

## AUTHOR CONTRIBUTIONS

Thomas W. Owen, Yujiang Wang, and Peter N. Taylor contributed to the conception and design of the study. Thomas W. Owen, Vytene Janiukstyte, Gerard R. Hall, Jonathan J. Horsley, Andrew McEvoy, Anna Miserocchi, Jane de Tisi, John S. Duncan, Fergus Rugg‐Gunn, Yujiang Wang, and Peter N. Taylor, contributed to the acquisition and analysis of data. Thomas W. Owen and Peter N. Taylor contributed to the drafting of the text and preparing the figures.

## CONFLICT OF INTEREST STATEMENT

No relevant conflicts of interest are reported. We confirm that we have read the Journal's position on issues involved in ethical publication and affirm that this report is consistent with those guidelines.

## Supporting information


Appendix S1

## Data Availability

Data and code to reproduce the figures are available upon reasonable request.
