# Supplementary material for: Identifying epileptogenic abnormalities through spatial clustering of MEG interictal band power
Source: Epilepsia Open. 2023 Jun 5;8(3):1151–6. doi: 10.1002/epi4.12767 (PMC10472397; doi:10.1002/epi4.12767)
Supplement: Supplementary file 1 — Appendix S1 [file EPI4-8-1151-s001.docx]

Identifying epileptogenic abnormalities through spatial clustering of MEG interictal band power

Thomas W. Owen^1^, Vytene Janiukstyte^1^, Gerard R. Hall^1^,
Jonathan J. Horsley^1^, Andrew McEvoy^3,5^,
Anna Miserocchi^3,5^, Jane de Tisi^3,4,5^, John S. Duncan^3,4,5^,
Fergus Rugg-Gunn^3,5^, Yujiang Wang^1,2,3,5^, Peter N. Taylor^1,2,3,5^

1. CNNP Lab (www.cnnp-lab.com), Interdisciplinary Computing and Complex BioSystems Group, School of Computing, Newcastle University, Newcastle upon Tyne, United Kingdom
2. Faculty of Medical Sciences, Newcastle University, Newcastle upon Tyne, United Kingdom
3. UCL Queen Square Institute of Neurology, Queen Square, London, WC1N 3BG, United Kingdom
4. NIHR University College London Hospitals Biomedical Research Centre, UCL Queen Square Institute of Neurology, London WC1N 3BG, United Kingdom
5. National Hospital for Neurology & Neurosurgery, Queen Square, London, WC1N 3BG, United Kingdom

* [t.w.owen1@newcastle.ac.uk](mailto:t.w.owen1@newcastle.ac.uk) & [peter.taylor@newcastle.ac.uk](mailto:peter.taylor@newcastle.ac.uk) Orcid ID 0000-0003-2144-9838

# Supplementary

## Analysis using other measures of overlap

One potential drawback of the dice score is that it penalises the degree of overlap based on both the false positive and false negative rate. That is, the degree of overlap is dampened if the actual resection extends beyond the abnormality cluster, or if the abnormality cluster extends to areas outside of the actual resection. We assess the degree of overlap between the abnormality cluster using alternative measures, namely the positive predictive value (PPV). The PPV measure neglects the effects of the false negative rate, capturing the ratio of the overlap (TP) relative to the total number of regions in either the abnormality cluster, or actual resection. We compare the degree of overlap between surgical outcome groups using (1) the PPV that quantifies the proportion of the abnormality cluster that intersects with the actual resection relative to all regions within the abnormality cluster, and (2) the PPV measuring the proportion of the actual resection within the abnormality cluster. Parameter values of K=3 and N=50 were used for all patients to directly compare with the dice score in main text (Figure [2](#results)). The results of the PPV measures studies are illustrated in Figure [3](#supp:ppv_measures). Comparable results to the dice score can be seen for both measures of the PPV. Similar AUC values to the dice score indicate that neither the actual resection, nor abnormality cluster extending into alternative regions have a negative impact on the separability of outcome groups. However, each measure provides a different perspective on the overlap, providing clinical teams with additional clarity and information during pre-surgical planning.


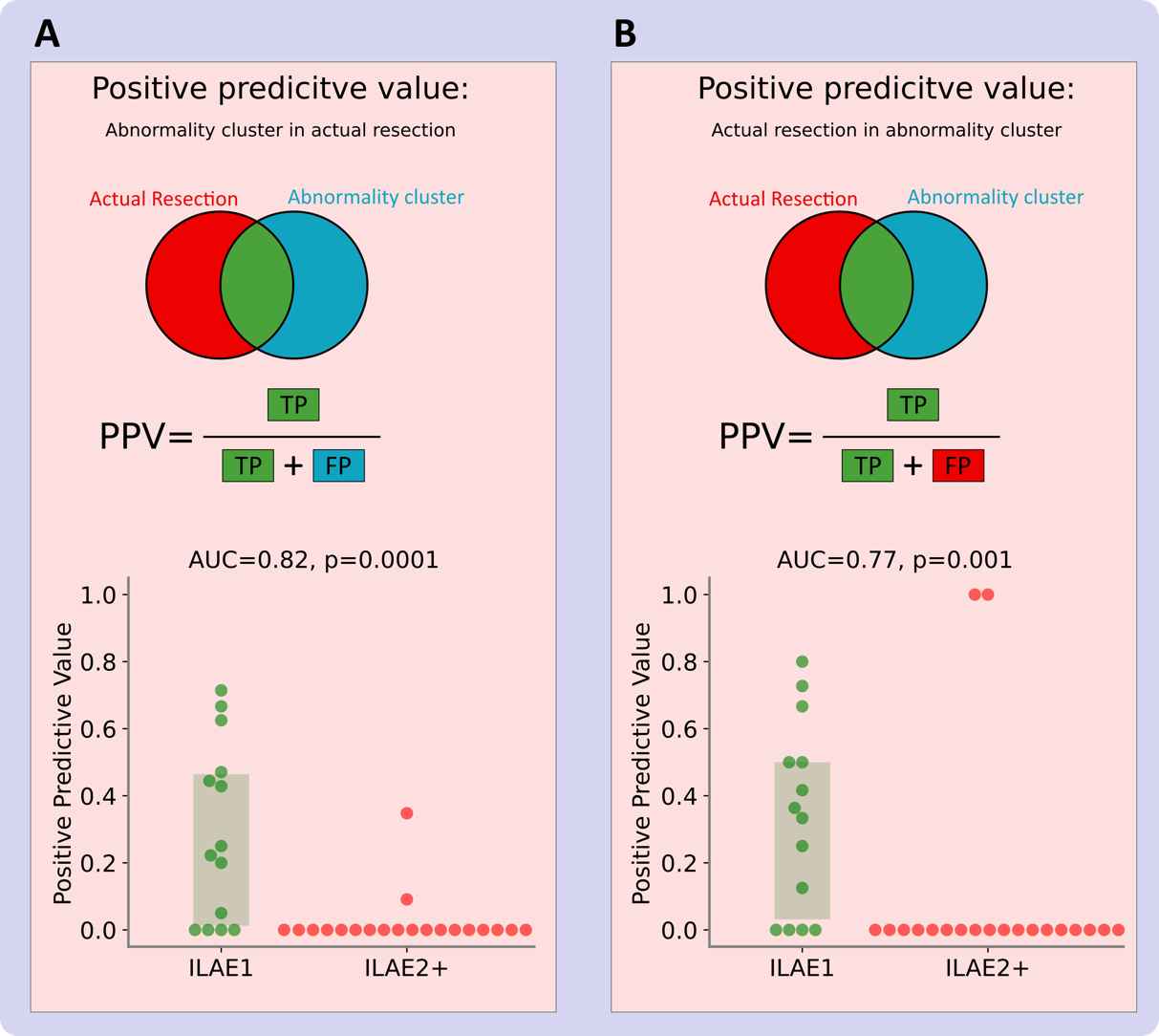


**Overlap between the abnormality cluster and actual resection using the positive predictive values.** The degree over overlap between the abnormality cluster and actual resection were compared across surgical outcome groups using the positive predictive value (PPV). **(A)** First we quantify how much of the abnormality cluster overlaps with the actual resection, thus accounting for proposed regions that extend beyond the true resection. The degree of overlap differed significantly between surgical outcome groups (AUC=0.82, p=0.0001). **(B)** The second PPV measure captures the proportion of the actual resection that overlaps with the abnormality cluster. This measure also accounts for the regions that were actually resected but not captured within the abnormality cluster. A significant separability of outcome groups was also detected (AUC=0.77, p=0.001) indicating that both measures could be leveraged to aid clinical decision making.

## Table of patient data

**Summary of patient metadata and clustering results.** Commonly acquired patient metadata are reported including the side of surgical resection, localisation of the resection (F: Frontal, T: Temporal, P: Parietal, O: Occipital), one year post-operative outcome, long term surgical outcome, and whether the patient relapsed at a later date. Additionally, the dice score, measuring the overlap between the abnormality cluster and actual resection is reported.

| Patient ID | Side | Resection Site | Surgical Outcome (1 year) | Surgical Outcome (Long term) | Years of follow up | Relapsed (Yes/No) | Dice Score |
| --- | --- | --- | --- | --- | --- | --- | --- |
| 1 | L | F | ILAE 2+ | ILAE 2+ | 5 | - | 0 |
| 2 | L | F | ILAE 2+ | ILAE 2+ | 5 | - | 0 |
| 3 | R | F | ILAE 2+ | ILAE 2+ | 5 | - | 0 |
| 4 | L | F | ILAE 1 | ILAE 1 | 2 | No | 0.308 |
| 5 | L | F | ILAE 1 | ILAE 2+ | 5 | Yes | 0.375 |
| 6 | R | F | ILAE 2+ | ILAE 1 | 5 | - | 0 |
| 7 | R | F | ILAE 2+ | ILAE 1 | 5 | - | 0.516 |
| 8 | L | F | ILAE 2+ | ILAE 2+ | 5 | - | 0 |
| 9 | R | P | ILAE 1 | ILAE 1 | 5 | No | 0 |
| 10 | L | F | ILAE 1 | ILAE 1 | 5 | No | 0.571 |
| 11 | L | F | ILAE 2+ | ILAE 2+ | 3 | - | 0 |
| 12 | R | T | ILAE 1 | ILAE 1 | 5 | No | 0 |
| 13 | L | F | ILAE 2+ | ILAE 2+ | 4 | - | 0 |
| 14 | R | P | ILAE 2+ | ILAE 1 | 5 | - | 0.364 |
| 15 | L | O | ILAE 1 | ILAE 2+ | 5 | Yes | 0 |
| 16 | R | F | ILAE 2+ | ILAE 2+ | 2 | - | 0 |
| 17 | R | F | ILAE 1 | ILAE 2+ | 5 | Yes | 0.556 |
| 18 | R | P | ILAE 2+ | ILAE 2+ | 5 | - | 0 |
| 19 | L | T | ILAE 1 | ILAE 1 | 5 | No | 0 |
| 20 | L | T | ILAE 2+ | ILAE 2+ | 5 | - | 0 |
| 21 | L | T | ILAE 2+ | ILAE 2+ | 5 | - | 0 |
| 22 | L | F | ILAE 1 | ILAE 1 | 5 | No | 0.308 |
| 23 | L | FP | ILAE 2+ | ILAE 2+ | 5 | - | 0.167 |
| 24 | R | F | ILAE 2+ | ILAE 2+ | 5 | - | 0 |
| 25 | L | F | ILAE 1 | ILAE 1 | 5 | No | 0.471 |
| 26 | L | F | ILAE 2+ | ILAE 2+ | 4 | - | 0 |
| 27 | L | F | ILAE 2+ | ILAE 2+ | 5 | - | 0 |
| 28 | R | OP | ILAE 1 | ILAE 1 | 5 | No | 0.25 |
| 29 | R | F | ILAE 1 | ILAE 1 | 5 | No | 0.571 |
| 30 | R | O | ILAE 1 | ILAE 2+ | 5 | Yes | 0.526 |
| 31 | L | OP | ILAE 2+ | ILAE 2+ | 5 | - | 0 |
| 32 | R | F | ILAE 2+ | ILAE 2+ | 4 | - | 0 |
| 33 | R | P | ILAE 2+ | ILAE 2+ | 5 | - | 0 |
| 34 | R | F | ILAE 1 | ILAE 2+ | 5 | Yes | 0.071 |
